# Gene expression in the dorsal root ganglion and the cerebrospinal fluid metabolome in polyneuropathy and opioid tolerance in rats

**DOI:** 10.1016/j.ibneur.2024.05.006

**Published:** 2024-05-24

**Authors:** Fredrik H.G. Ahlström, Hanna Viisanen, Leena Karhinen, Vidya Velagapudi, Kim J. Blomqvist, Tuomas O. Lilius, Pekka V. Rauhala, Eija A. Kalso

**Affiliations:** a Department of Pharmacology, Faculty of Medicine, Biomedicum 1, University of Helsinki, Haartmaninkatu 8, 00014, Finland; b Individualized Drug Therapy Research Programme, Faculty of Medicine, Biomedicum 1, University of Helsinki, Haartmaninkatu 8, 00014, Finland; c Metabolomics Unit, Institute for Molecular Medicine Finland FIMM, University of Helsinki, P.O. Box 20, FI-00014, Finland; d Department of Clinical Pharmacology, University of Helsinki and Helsinki University Hospital, Tukholmankatu 8C, 00014, Finland; e Department of Emergency Medicine and Services, University of Helsinki and HUS Helsinki University Hospital, Haartmaninkatu 4, Helsinki 00290, Finland; f SleepWell Research Programme, Faculty of Medicine, , University of Helsinki, Haartmaninkatu 3, 00014, Finland; g Department of Anaesthesiology and Intensive Care Medicine, Helsinki University Hospital and University of Helsinki, HUS, Stenbäckinkatu 9, P.O. Box 440, 00029, Finland

**Keywords:** Neuropathic pain, opioid tolerance, dorsal root ganglion, cerebrospinal fluid

## Abstract

First-line pharmacotherapy for peripheral neuropathic pain (NP) of diverse pathophysiology consists of antidepressants and gabapentinoids, but only a minority achieve sufficient analgesia with these drugs. Opioids are considered third-line analgesics in NP due to potential severe and unpredictable adverse effects in long-term use. Also, opioid tolerance and NP may have shared mechanisms, raising further concerns about opioid use in NP. We set out to further elucidate possible shared and separate mechanisms after chronic morphine treatment and oxaliplatin-induced and diabetic polyneuropathies, and to identify potential diagnostic markers and therapeutic targets. We analysed thermal nociceptive behaviour, the transcriptome of dorsal root ganglia (DRG) and the metabolome of cerebrospinal fluid (CSF) in these three conditions, in rats. Several genes were differentially expressed, most following oxaliplatin and least after chronic morphine treatment, compared with saline-treated rats. A few genes were differentially expressed in the DRGs in all three models (e.g. *Csf3r* and *Fkbp5*). Some, e.g. *Alox15 and Slc12a5,* were differentially expressed in both diabetic and oxaliplatin models. Other differentially expressed genes were associated with nociception, inflammation, and glial cells. The CSF metabolome was most significantly affected in the diabetic rats. Interestingly, we saw changes in nicotinamide metabolism, which has been associated with opioid addiction and withdrawal, in the CSF of morphine-tolerant rats. Our results offer new hypotheses for the pathophysiology and treatment of NP and opioid tolerance. In particular, the role of nicotinamide metabolism in opioid addiction deserves further study.

## Highlights

• *Fkbp5 and Csf3r* were differentially expressed in all models.
• Many genes related to nociception and inflammation were affected by the treatments.
• Our study is the first to characterize the CSF metabolome in these animal models.
• Nicotinamide levels in the CSF rose greatly following chronic morphine treatment.
• Nicotinamide is thought to be of importance in opioid addiction and withdrawal.

## 1. Introduction

Painful neuropathies are often refractory to the recommended pharmacotherapy (Bouhassira et al., 2008, Finnerup et al., 2015, Torrance et al., 2006). The same drugs (antidepressants and gabapentinoids) are used in different conditions even though their pathophysiologies may significantly differ (Bannister et al., 2020: Finnerup et al., 2021). Opioids are considered a third-line option for neuropathic pain (NP) due to problems in long-term use, e.g. opioid tolerance (Sommer et al., 2020; Paul et al., 2021). Interestingly, the development of opioid tolerance and NP may share some mechanisms (Kadhim et al., 2018, Mayer et al., 1999). This raises questions about the possible detrimental effects of opioids on underlying neuropathies.

We studied oxaliplatin-induced and diabetic polyneuropathies and opioid tolerance with transcriptomics and metabolomics to elucidate their molecular mechanisms (Aderemi et al., 2021, Feldman et al., 2019, Hrdlickova et al., 2017; Miltenburg and Boogerd, 2014; Morgan and Christie, 2011; Weickhardt et al., 2011). We focused on dorsal root ganglia (DRG), which have a seminal role for the well-being of peripheral nerves, and on cerebrospinal fluid (CSF), which offers a clinically available opportunity for biomarker diagnostics.

DRGs comprise the somata of primary nociceptors and glial cells, and are an essential site for the modulation of nociception. Injury to nociceptors causes release of cytokines and growth factors, and changes in gene expression. Schwann cells and macrophages further regulate this inflammation, leading to sensitization in the DRG and central nervous system (CNS) (Balakrishnan et al., 2020; Barker et al., 2020; Black et al., 1999; Boucher and McMahon, 2001; Catala and Kubis, 2013; Fregnan et al., 2012; Yu et al., 2020). So far, most research on changes in gene expression in neuropathies has focused on traumatic peripheral nerve injury (Gu et al., 2016, Pokhilko et al., 2020, Uttam et al., 2018).

As sampling of CSF is feasible, it could be used to develop diagnostic and prognostic CSF biomarkers for many pathological conditions (Krause et al., 2021). Methods to analyse the metabolome of the CSF have only recently been described (Clish, 2015, Johnson et al., 2016). Thus far, no studies have characterized the metabolome of CSF in neuropathy or following chronic opioid exposure.

Prolonged use of opioids can lead to analgesic tolerance (Morgan and Christie, 2011; Mercadante et al., 2019) and increased doses are associated with serious adverse effects. Several mechanisms are involved in opioid tolerance (Bohn et al., 1999, Ferrini et al., 2013, Raghavendra et al., 2002, Reiss et al., 2022, Williams et al., 2013). In cultured DRGs, morphine has been shown to induce calcitonin gene-related peptide and substance P expression (Ma et al., 2000), suggesting a role for the DRG in modulating responses to chronic opioid exposure.

Oxaliplatin is a cytotoxic drug commonly used in colorectal cancer. It arrests cell division by creating platinum-DNA adducts. It typically causes cold allodynia, paraesthesia, and dysaesthesia (Weickhardt et al., 2011). Mechanisms causing oxaliplatin-induced neuropathy may involve voltage-gated sodium channels, transient receptor potential channels, and platinum accumulation in neural tissue (Cavaletti et al., 2001, Park et al., 2009). Oxaliplatin has been reported to cause global decreases in gene expression (Yan et al., 2015). Interestingly, high CSF levels of oxaliplatin are associated with the development of the neuropathy (Huang et al., 2016).

Diabetic neuropathy is characterized by autonomic and peripheral nervous system dysfunction, often painful. Diabetic neuropathy is caused by microvascular damage with consequent compromised neural blood supply (Forbes and Cooper, 2013). Oxidative stress, mitochondrial dysfunction, apoptosis, and immune responses have been reported in the DRGs of rats with streptozotocin (STZ)-induced diabetes (Schmeichel et al., 2003, Ton et al., 2013, Rahman et al., 2016), and the changes in gene expression in the DRG of STZ-rats have been studied (Guo et al., 2018, Zhu et al., 2008)

We investigated the DRG transcriptome and CSF metabolome in two polyneuropathies and in opioid tolerance, aiming to better understand their pathophysiologies, and to generate new treatment targets and potential diagnostic biomarkers in the CSF.

## 2. Material and methods

### 2.1. Study design

**Fig. 1:**
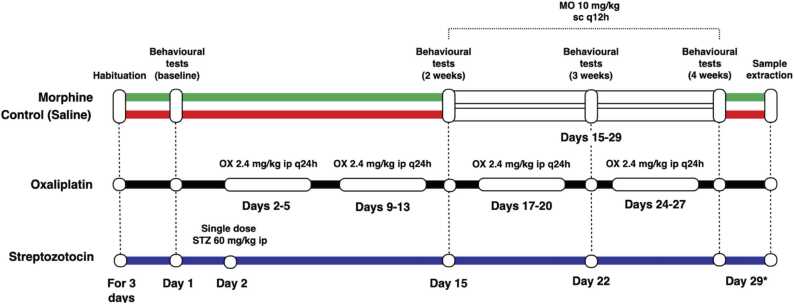
Design of the study. Timeline for administration of morphine (sc) with saline control group, oxaliplatin (ip) and streptozotocin (ip) treatments. Behavioural tests were conducted before treatments (baseline) and after 2, 3 and 4 weeks from the start of the experiment. The behavioural tests were performed in the morphine and saline groups on Days 15, 22, and 29, both before and 1 hour after administration of the drugs. *Samples were extracted on Days 29–31, with morphine and saline treatments continuing until 12 h before sample extraction. Abbreviations: MO = morphine; OX = oxaliplatin; STZ = streptozotocin; sc = subcutaneous; ip = intraperitoneal; q12h = every 12 hours; q24h = once daily.

In this study, we studied three models of neuropathy in rats, by administering morphine, oxaliplatin and streptozotocin, the protocol is described in Fig. 1 and below.

### 2.2. Animals

This study made use of 44 male Sprague-Dawley rats, weighing approximately 200–230 g at the time of the first behavioural tests. The animals were kept at the Laboratory Animal Centre of Biomedicum Helsinki, with two animals per cage. A standard diet of food and water was provided ad libitum. The facility had alternating 12-hour-long light and dark conditions. All groups received similar physical handling, regardless of pharmacological treatment. The cages of the diabetic rats were changed daily. Prior to the behavioural tests, the animals were habituated to the testing conditions for three days. Utmost care was taken to minimize stress caused by the experiments. The Regional State Administrative Agency for Southern Finland approved all experimental plans and procedures. We followed the regulations described in the EU directive “Act on the Protection of Animals Used for Scientific or Educational Purposes” (497/2013), and adhered to the ARRIVE 2.0 guidelines (Percie du Sert et al., 2020) and the 3Rs principle (Russell and Burch, 1959).

### 2.3. Animal models

We allocated the animals to the following groups: one receiving subcutaneous (sc) morphine injections (n = 10), one receiving a similar regimen of saline injections (n = 10), one receiving the injections of the cytotoxic agent oxaliplatin (n = 10), and a fourth group, in which diabetes was induced with streptozotocin (n = 14). The first two groups were blinded and the saline-treated group served as the control group for all comparisons. For safety concerns and practical reasons, the animals injected with oxaliplatin and streptozotocin were not blinded to the injecting investigator. The treatment status of the morphine and saline groups was revealed after the final behavioural tests.

#### 2.3.1. Opioid tolerance

The induction of morphine tolerance was initiated with injections of morphine after the baseline tests on Day 15. Injections of sc morphine hydrochloride at doses of 10 mg/kg of morphine base in a volume of 1 ml/kg were administered twice daily, twelve hours apart, for 14–16 days, depending on the sample extraction day. The last dose was administered twelve hours before the isolation of samples. The extraction date varied, as it was not feasible to extract all samples on the same day. The timeline of the treatments is illustrated in Fig. 1.

#### 2.3.2. Control group

The control group consisted of rats receiving saline injections (1 ml/kg) sc using the same protocol as for the morphine-treated rats.

#### 2.3.3. Oxaliplatin-related neuropathy

Oxaliplatin-related neuropathy was induced by administering ip oxaliplatin, starting on the day after the first baseline behavioural tests (Day 2). Oxaliplatin injections of 2.4 mg/kg, diluted with a 5% solution of glucose, with an injection volume of 2 ml/kg, were administered on four consecutive days, after which no injections were given for three days. This pattern was repeated during four consecutive weeks, yielding a total accumulated dose of oxaliplatin of 38.4 mg/kg. This administration schedule was comparable to previously used protocols (Cavaletti et al., 2001).

#### 2.3.4. Streptozotocin-induced diabetes

A single injection of ip streptozotocin 60 mg/kg was administered to induce insulin-dependent diabetes mellitus in rats on the day after the first baseline behavioural tests (Day 2) (Akbarzadeh et al., 2007). To confirm the development of diabetes mellitus, blood glucose levels were measured before the injection, and three days and four weeks after the injection.

### 2.4. Behavioural tests

To determine changes in behaviour due to the treatments, we used the acetone evaporation (Vissers and Meert, 2005), hot plate (Woolfe and McDonald, 1944), and tail-flick tests (D'Amour and Smith, 1941). These were performed before starting the treatments (Day 1), and after, three and four weeks (Days 15, 22 and 29–31). Starting from Day 15, hot plate testing was also included in the morphine and saline groups.

Development of antinociceptive tolerance to morphine was confirmed with the tail-flick and the hot plate tests.

#### 2.4.1. Acetone evaporation test

Cold allodynia was assessed using acetone which was applied on one hind paw, after which the animal was monitored for one minute. A positive response was recorded if the animal attended to its paw, signalling a positive response. After monitoring, a minute-long break followed, after which the test was repeated for a total of five sprays per animal. Results are reported as the number of positive responses.

#### 2.4.2. Tail-flick test

Heat nociception was assessed with the tail-flick test. The animals were habituated for 10 minutes in Plexiglas cylinders before undergoing three rounds of testing, after which one mean result was calculated for each animal. In the morphine and saline group, testing was done before and one hour after drug administration. Testing was conducted with a Ugo Basile Tail-Flick 37360 apparatus (Gemonio, Italy). The intensity was set to 5.5 and a cutoff time of 10 seconds was used to avoid tissue damage.

#### 2.4.3. Hot plate test

A single round of hot plate testing was conducted before and one hour after morphine administration using a Ugo Basile Hot / Cold Plate 35100 (Gemonio, Italy), with the temperature set at 52 °C, using a cutoff time of 60 seconds. When discomfort, signalled by forceful retraction of the hind paw or equivalent reaction, was observed, the test ended and the time was recorded.

### 2.5. Sample collection

The animals were euthanized using transcardial perfusion with a phosphate buffer solution, after having been anaesthetized with a 2–4% isoflurane inhalation.

Dorsal root ganglia were excised after the fourth week behavioural tests, twelve hours after the last morphine and saline injections in these groups. The ganglia were acquired bilaterally from segments L4 and L5. All four DRGs from one animal were pooled into one sample.

Following the collection of samples, depending on the extraction day, the samples were either frozen directly to -70 °C, or stored in an RNAlater solution at 4 °C.

We also collected CSF samples (ca 100 μl/sample) as previously described (Nirogi et al., 2009), and done by our group (Blomqvist et al., 2022). The procedure was carried out under 2% isoflurane anaesthesia. First, the rat’s neck fur was cut, after which the rat was secured in a stereotaxic frame with its head tilted downward at about 45°, making the area between the occipital protuberance and the atlas spine more prominent. The overlying skin was swabbed with 70% ethanol, after which CSF was drawn from the cisterna magna using a syringe with a horizontally inserted needle, without any incision. As the needle entered, changes in resistance were noted, and gentle aspiration yielded CSF.

### 2.6. RNA extraction and sequencing

The extraction and sequencing of RNA from the dorsal root ganglia were conducted at the Functional Genomics Unit of the Faculty of Medicine (FuGU), University of Helsinki. Six pooled DRG samples per group were sequenced. Extraction was done using Precellys soft-tissue beads and Trizol, and purification of samples was conducted using Qiagen’s RNeasy Mini Kit. Sample quality was assessed with Bioanalyzer RNA and DNA quality control assays, and deemed sufficient, with total RNA RIN values ranging from 7.1 to 8.5. rRNA-depletion and library preparation were carried out with Illumina ScriptSeq Complete Gold Kit. Sequencing was done using two Illumina NextSeq 500/550 High Output 1×75 bp kits.

### 2.7. CSF metabolome extraction and analysis

CSF samples were stored at -80 °C until analysed. CSF metabolomic analyses were carried out at the Institute for Molecular Medicine Finland Metabolomics Unit (FIMM) using targeted liquid chromatography mass spectrometry (LC/MS). Common polar, non-ionic metabolites, including amino acids, bile acids, choline metabolites, carbohydrates, enzyme cofactors, nucleosides, and nucleobases (n = 102), were targeted using the BioCrates p180 kit, which has standards for isotopic quantification of these metabolites. Metabolite profiling analysis of the samples was performed using liquid chromatography-mass spectrometry. Briefly, 10 µL of labeled internal standard mixture was added to the 100 µL of samples, and the samples were allowed to equilibrate with the internal standards. A total of 400 µL of extraction solvent (1% formic acid in acetonitrile) was added and the collected supernatant was dispensed into an OstroTM 96-well plate (Waters Corporation, Milford, USA) and then filtered by applying a vacuum at a delta pressure of 300–400 mbar for 2.5 minutes on a Hamilton robot's vacuum station. After this, 5 μL of filtered sample extract was injected into an Acquity UPLC system coupled to a Xevo® TQ-S triple quadrupole mass spectrometer (Waters Corporation, Milford, MA, USA), which was operated in both positive and negative polarities with a polarity switching time of 20 msec for metabolite separation and quantification. The Multiple Reaction Monitoring (MRM) acquisition mode was selected for the quantification of metabolites. MassLynx 4.1 software was used for data acquisition, data handling, and instrument control. The data were processed using TargetLynx software (Nandania et al., 2018). Of the 102 targeted metabolites, 86 had less than 50% missing values within a group and were included in the subsequent data analysis.

### 2.8. Statistical analyses

All statistical analyses for the behavioural data were performed using GraphPad Prism 9 (La Jolla, CA, USA). Statistical significances, where applicable, were determined using Sidak multiple comparison-corrected 2-way ANOVA analyses.

The transcriptome was analysed using the open-source software Chipster v3.10. Quality reports for the FASTQ-files were generated using the FastQC package (Wingett and Andrews, 2018). Alignment to the Ensembl Rattus norvegicus 6.0.86 genome was done using the TopHat2 package (Kim et al., 2013) for single-ended data with default parameters, except for using the secondstrand setting, as appropriate. Count tables were built using the HTSeq package (Anders et al., 2015). Differential expression was calculated using the EdgeR package (Robinson et al., 2010) and default parameters with the adjusted p-value criterion being < 0.05. In the oxaliplatin group, the expression of some genes was greatly upregulated in one rat only; these genes were largely related to muscle tissue and were omitted from subsequent analyses when the number of transcripts was >3 larger than in any other rat. Volcano plots were made using VolcaNoseR (Goedhart and Luijsterburg, 2020). Clustering of significantly up- and downregulated genes was performed using Gene Cluster 3.0 software and heatmap visualizations were produced using Java TreeView 1.16r4 software, as previously published Lindford et al., 2021).

Pathway analyses were performed with Advaitas iPathwayGuide (Nguyen et al., 2019) using FDR adjusted p-value < 0.05 and log_2_ fold change > 0.6 as input criteria for the EdgeR analysis. Pathway analysis results were corrected using FDR. Gene Ontology terms, such as biological processes, were corrected using Elim Pruning. The pathway analyses of the three models were then joined together using iPathwayGuide, yielding a meta-analysis of results from the different pain models to identify similarities in the differential expression of the genes. We defined the term ‘glial cell-related changes’ as alterations in the expression of either a single gene or several genes known to be good markers for PNS glial cells or their function, or biological processes found in the Gene Ontology Consortium Database that contained the keyword “glia”. Likewise, pain-related changes were defined as single gene expression changes in genes included in the list built by Pain Networks or Biological processes containing the word “pain”.

The metabolome of extracted CSF was analysed in the FIMM metabolomics unit using MetaboAnalyst (V 3.5) software (Xia and Wishart, 2016). Single metabolite analysis was conducted using pairwise t-tests.

## 3. Results

### 3.1. The effect of oxaliplatin and streptozotocin treatment on acetone and tail-flick tests

**Fig. 2:**
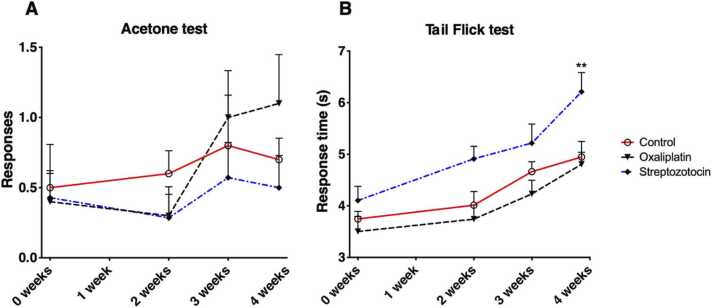
Results of the acetone test for cold allodynia (A). The mean number of responses out of five acetone sprays and standard errors of measurement (SEMs) are reported. Results of the tail-flick tests for thermal hyperalgesia (B). Testing was carried out on Days 1, 15, 22 and 29 (Fig. 1). The mean response times and SEMs are reported. The cutoff time was 10 seconds in the tail-flick test. The number of animals was 14 in the STZ group and 10 in the other groups. A two-way ANOVA was used; ** = p < 0.01.

In the acetone test, there was an upward trend in the responses in the oxaliplatin-treated group, though this was statistically insignificant (p > 0.05) (Fig. 2). Tail-flick response times showed a significant increase after 4 weeks in the STZ group (p = 0.0159). Other tail-flick results showed no significant changes.

### 3.2. Development of opioid tolerance

**Fig. 3:**
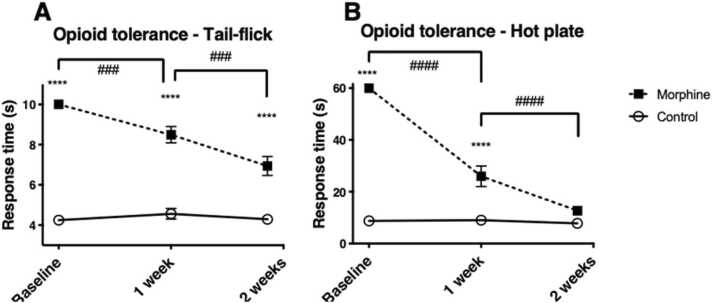
Development of opioid tolerance measured using tail-flick (A) and hot plate (B) tests one hour after 10 mg/kg of subcutaneous morphine injections. Testing was carried out before the first morphine and saline injections (baseline), and after one and two weeks of chronic administration (Days 22 and 29 in Fig. 1). Mean response times are reported with SEMs (n = 10). The cutoff times were 10 s in the tail-flick and 60 s in the hot plate tests. Two-way ANOVA; **** = p < 0.0001.

Morphine caused significant antinociception in the opioid-naïve rats. After two weeks of treatment, the behavioural responses in the morphine-treated group did not differ from those in the saline control group one hour after injections in the hot plate test, confirming development of morphine tolerance. After two weeks, the tail-flick test still demonstrated a significant, albeit much smaller, difference in response to acute morphine (p < 0.0001). (Fig. 3).

### 3.3. Blood glucose and weight

The mean blood glucose level before the STZ injections was 6.85 mmol/l and all but one rat had a blood glucose of > 33 mmol/l three days after the STZ injection, with one rat having a blood glucose of 27.3 mmol/l; results at the time of sample extraction were identical.

The weight gain was decreased in all treatment groups, compared with the control group (p < 0.0001).

### 3.4. Differential gene expression in DRG

**Fig. 4:**
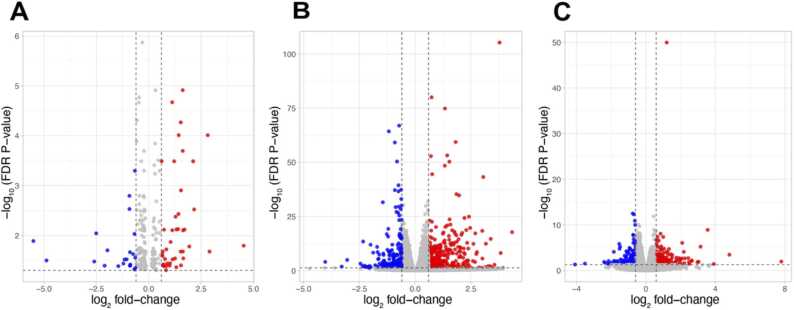
Volcano plots reporting log_2_ fold-changes and -log_10_ of FDR-corrected p-values (-log(FDR)) following morphine (A), oxaliplatin (B) and streptozotocin (C) treatments. The dotted lines are at FDR = 0.05 on the x-axis and at log_2_ fold-change -0.6 and 0.6 on the y-axis. EdgeR was utilized for DE analysis. n = 6 in all groups.

**Fig. 5:**
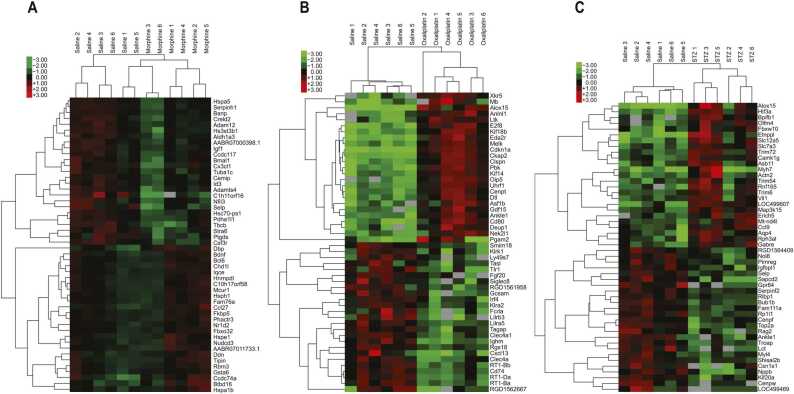
Heat map data of 25 most up- and downregulated genes after morphine (A), oxaliplatin (B) and streptozotocin (C) treatments. FDR < 0.05 in all genes. EdgeR was utilized for DE analysis.

**Fig. 6:**
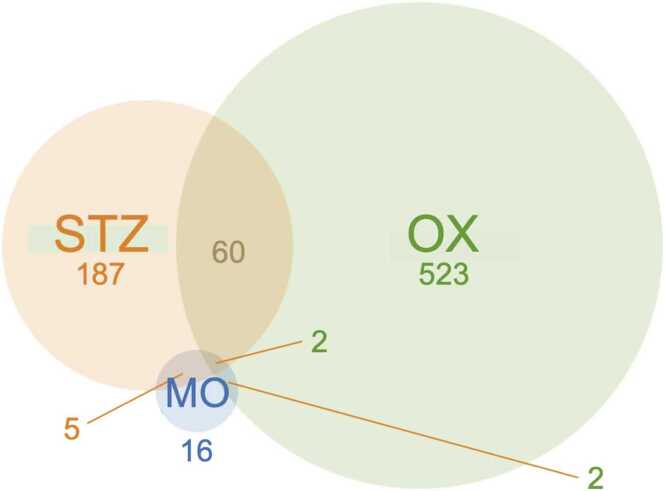
Venn Diagram demonstrating the number of differentially expressed genes in each model. Differential expression analyses were conducted using EdgeR. Log_2_-FC > 0.6 FDR < 0.05; n = 6 in all groups; MO = Morphine; OX = Oxaliplatin; STZ = Streptozotocin.

**Fig. 7:**
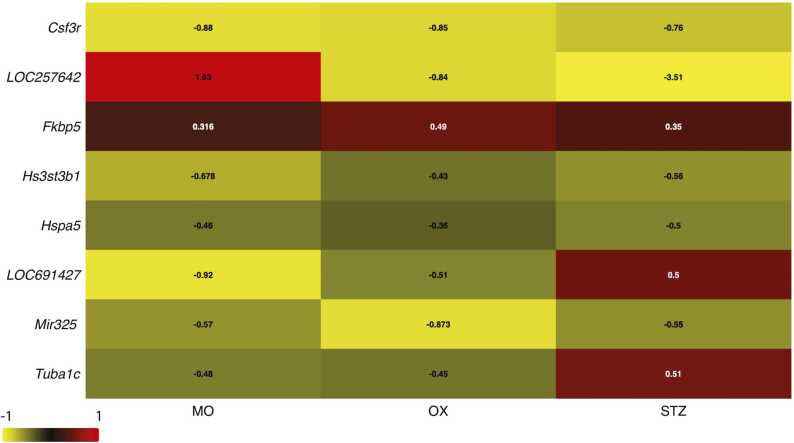
Heatmap of gene expression changes common to all models. Differential gene.

The morphine-, oxaliplatin-, and STZ-treated animals showed 228, 4364 and 1687 differentially expressed genes, compared with the saline (control) group, respectively, using the EdgeR package (FDR < 0.05). The differential gene expression data of all genes with FDR < 0.05 is found in supplementary data file 1. Fig. 4, Fig. 5 demonstrate the differential gene expression in Volcano plots and the most up- and downregulated genes in heat maps, respectively. Fig. 6 illustrates the number of common differentially expressed genes in the different treatment groups using Venn diagrams. Two transcripts, *Csf3r* and *LOC257642*, were markedly differentially expressed (log_2_-FC > 0.6) in all three models (Fig. 7). For *Csf3r*, expression decreased in all models, the log_2_-FCs for the morphine, oxaliplatin and STZ groups being -0.88, -0.85 and -0.76, respectively. Lower log_2_-FCs (> 0.3) were common in all groups for *Fkbp5*, *Hs3st3b1*, *Hspa5*, *LOC691427*, *Mir325,* and *Tuba1c*. Five genes showed changes in the same direction in all groups: *Fkbp5* was upregulated whereas *Csf3r, Hs3st3b1*, *Hspa5* and *Mir325* were downregulated (Fig. 7). In addition, *Alox15*, *Slc12a5*, and *Gabrb1* showed markedly increased expression in the oxaliplatin and STZ groups, the log_2_-FCs being 4.34 and 3.56 (*Alox15*), 1.68 and 2.18 (*Slc12a5*), and 1.02 and 1.22 (*Gabrb1*), respectively. *Nppb* was downregulated in the oxaliplatin and STZ groups (log_2_-FCs -1.19 and -1.85). *Dbp* expression was markedly increased in the morphine and STZ groups (log_2_-FCs 1.12 and 0.881).

expression (log2 fold-change > 0.3 and FDR BH-adjusted p-value < 0.05 in all gebes) was

calculated using the EdgeR package; log2 fold-change reported; n = 6 in all groups.

Abbreviations: MO = morphine tolerance; OX = oxaliplatin -related neuropathy; STZ =

streptozotocin -induced diabetic neuropathy.

#### 3.4.1. Glial cell-related differential gene expression

Six and seven genes whose expression is attributable to glial cell presence or activity showed differential expression in the oxaliplatin and streptozotocin groups, respectively. *Gfap* expression was altered in both the OX and STZ models, but in different directions. Several genes coding for members of the s100 family of proteins also showed changes in expression in both models, as did the genes coding for HMG-CoA isoenzymes 1 and 2. (Table 1). In the morphine group, no glial cell-related genes showed altered expression.

**Table 1:** Glia-related changes in gene expression. Gene differential expression (log_2_-FC > 0.3 and FDR BH-adjusted p-values < 0.05) calculated using the EdgeR package; n = 6 in all groups. Common genes bolded.

**Oxaliplatin -related neuropathy**			**Streptozotocin -induced diabetic neuropathy**		
**Gene symbol**	**log**_**2**_**-FC**	**FDR**	**Gene symbol**	**log**_**2**_**-FC**	**FDR**
*Hmgcs1*	-0.51	2.52E-14	*Hmgcs2*	0.59	2.56E-09
*Fabp7*	-0.51	3.55E-07	*Dhh*	-0.58	1.67E-05
***Gfap***	0.63	2.03E-04	*Fabp7*	-0.54	7.82E-05
*S100a13*	-0.40	5.51E-04	*S100pbp*	0.32	4.56E-04
*S100g*	-1.32	5.95E-04	***Gfap***	-0.88	1.21E-03
*Egr2*	0.33	3.37E-02	*S100a16*	-0.37	5.84E-03
			*S100a5*	0.93	3.80E-02

#### 3.4.2. Changes in gene expression in pain-related genes

Pain-related changes were identified using the PainNetworks rat-centric list of pain genes (Perkins et al., 2013). Two pain-related transcripts were differentially expressed in the morphine group, *Ptgds* and *Bdnf*. In the oxaliplatin group, 30 pain genes showed altered expression, the most notable being *Chrna5*, *Ptpn6* and *RT1-Bb*. Finally, in the streptozotocin group, there were 34 pain-related genes with significant changes in expression, of which *Slc12a5*, *Aqp4* and *Grin2a* were the most differentially expressed ones (Table 2).

**Table 2:** Changes in gene expression in the pain-related genes (PainNetworks). Differential gene expression (log_2_-FC > 0.3 and FDR BH-adjusted p-value < 0.05) was calculated using the EdgeR package; n = 6 in all groups; FDR = False Discovery Rate.

**Morphine tolerance**					**Streptozotocin-induced diabetic polyneuropathy**		
**Symbol**	**Log**_**2**_**-FC**	**FDR**			**Symbol**	**Log**_**2**_**-FC**	**FDR**
*Bdnf*	0.370	2.07E-03			*Gal*	*-0.92*	*1.60E-05*
*Ptgds*	*-0.88*	*4.00E-02*			*Gfap*	*-0.88*	*1.21E-03*
**Oxaliplatin -related neuropathy**					*Edn1*	*-0.87*	*8.34E-03*
**Symbol**	**Log**_**2**_**-FC**	**FDR**			*Col9a1*	*-0.85*	*3.50E-03*
Ptpn6	*-1.73*	*1.12E-02*			*Trpa1*	*-0.64*	*1.24E-11*
RT1-Bb	*-1.64*	*3.04E-06*			*Rgs4*	-0.47	1.52E-03
Zeb2	*-1.19*	*2.84E-03*			*Ptger4*	-0.46	2.06E-02
Cd4	*-1.04*	*3.01E-10*			*Slc15a2*	-0.45	2.64E-04
Cybb	*-0.78*	*1.04E-05*			*Ptprz1*	-0.44	4.11E-08
Ednrb	*-0.71*	*4.57E-24*			*Htr2a*	-0.44	6.94E-03
Chrnb4	*-0.68*	*1.65E-02*			*Mme*	-0.43	1.55E-03
Gla	-0.55	2.93E-17			*Bdnf*	-0.41	4.39E+05
Lpar1	-0.41	2.35E-13			*Calca*	-0.41	4.75E-04
Lgals1	-0.41	1.66E-07			*S1pr3*	-0.40	5.21E-05
Gabrb3	-0.39	3.72E-02			*Adamts5*	-0.35	1.33E-04
Comt	-0.37	1.12E-16			*Ednrb*	-0.35	2.92E-04
Anxa1	-0.36	1.84E-05			*Nptx1*	-0.33	1.20E-07
Nlgn2	0.30	1.89E-03			*Tyrp1*	-0.32	5.03E+06
Nbl1	0.31	3.26E-06			*Adcyap1*	-0.32	1.24E-02
Dlg4	0.32	5.03E-14			*Ptn*	-0.31	4.41E-05
Grin1	0.32	1.23E-08			*Sparc*	-0.31	2.03E-03
Gnao1	0.33	3.72E-08			*Grin1*	0.30	2.69E-04
Npepps	0.34	2.88E-10			*Nbl1*	0.30	8.03E-05
Cacnb3	0.35	2.11E-13			*Ptgfr*	0.33	5.06E-03
Cdk5r1	0.37	4.79E-08			*Cnr1*	0.41	6.29E-06
Prkar1b	0.39	1.21E-11			*Kcnk3*	0.51	8.92E-03
Stx1a	0.39	2.26E-06			Adcyap1r1	0.52	1.95E-03
Csk	0.43	1.45E-08			Chrna5	0.57	2.94E-04
Cd274	0.43	2.01E-07			*Per1*	*0.69*	*6.66E-04*
Rgs4	0.44	4.10E-03			*Grm5*	*0.93*	*4.22E-02*
Il18	0.50	8.46E-03			*Klf7*	*0.95*	*4.56E-03*
Lpar3	0.55	9.25E-03			*Grin2a*	*1.15*	*1.49E-03*
Scn2b	0.57	8.87E-13			*Aqp4*	*1.30*	*4.58E-03*
*Gfap*	*0.63*	*2.03E-04*			*Slc12a5*	*2.18*	*8.03E-05*
*Chrna5*	*3.43*	*2.87E-02*					

#### 3.4.3. Analysis of biological processes

**Fig. 8:**
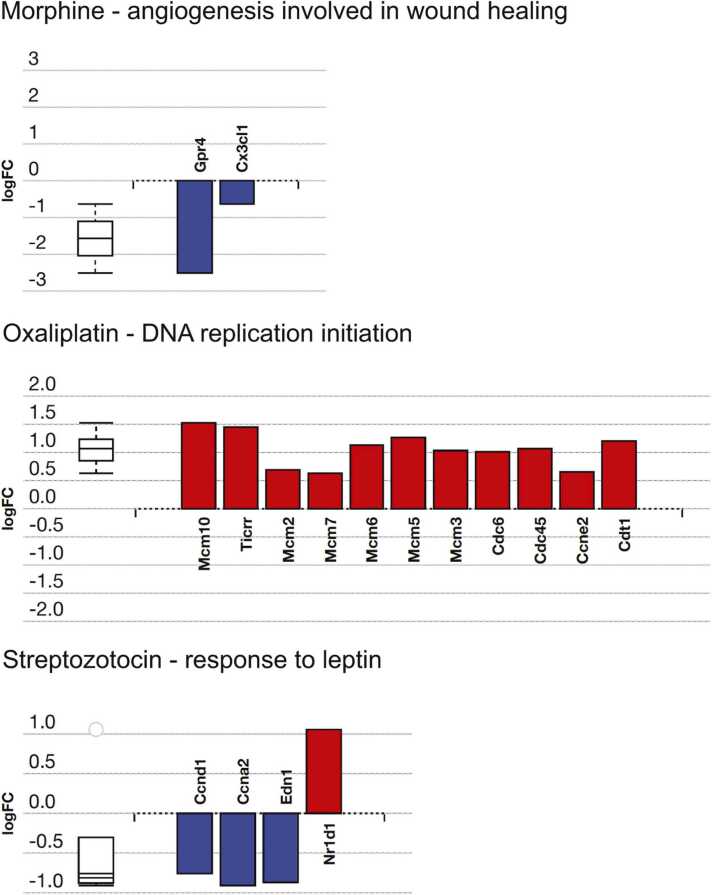
Differential expression in the most impacted biological process in the morphine, oxaliplatin and streptozotocin treatment groups. All genes exhibiting differential expression are ordered according to the statistical significance. The box-and-whisker plot to the left provides a summary of the distribution for all differentially expressed genes associated with this Gene Ontology (GO) term. The plot delineates the first quartile, median, and third quartile within the box, with outliers indicated by circle symbols. Figures are generated by AdvaitaBio’s iPathwayGuide.

The number of biological processes affected by treatment with morphine, oxaliplatin and streptozotocin were 69, 301 and 180 respectively, after Elim pruning (Table 3). Out of these, one process, DNA replication-dependent nucleosome assembly, was affected in all models. The genes affected in the most significantly impacted biologic process in each treatment group is shown in Fig. 8.

**Table 3:** Top five most affected biological processes. Analyses conducted using AdvaitaBio’s iPathway guide. All single gene results imputed, input criteria log_2_-FC > 0.6, FDR < 0.05. Top biological processes defined as those with the lowest p-value with Elim-pruning.

Top 5 Biological processes implicated - Morphine vs. Saline			
**Pruning Type: Elim**			
**GO Term**	**p-value**		
angiogenesis involved in wound healing	1.70E-04		
DNA replication-dependent nucleosome assembly	3.30E-04		
protein heterotetramerization	5.00E-04		
negative regulation of extrinsic apoptotic signalling pathway in the absence of a ligand	5.90E-04		
positive regulation of calcium-independent cell-cell adhesion	0.002		
Top 5 Biological processes implicated - Oxaliplatin vs. Saline			
**Pruning Type: Elim**			
**GO Term**	**p-value**		
DNA replication initiation	1.20E-08		
mitotic nuclear division	5.70E-07		
mitotic metaphase plate congression	8.60E-06		
cell division	9.00E-06		
mitotic cytokinesis	1.20E-05		
Top 5 Biological processes implicated - Streptozotocin vs. Saline			
**Pruning Type: Elim**			
**GO Term**	**p-value**		
response to leptin	1.50E-04		
transepithelial chloride transport	1.50E-04		
G protein-coupled purinergic nucleotide receptor signalling pathway	4.90E-04		
glycerol transport	8.10E-04		
cellular response to calcium ion	0.001

### 3.5. Metabolome of the CSF

**Fig. 9:**
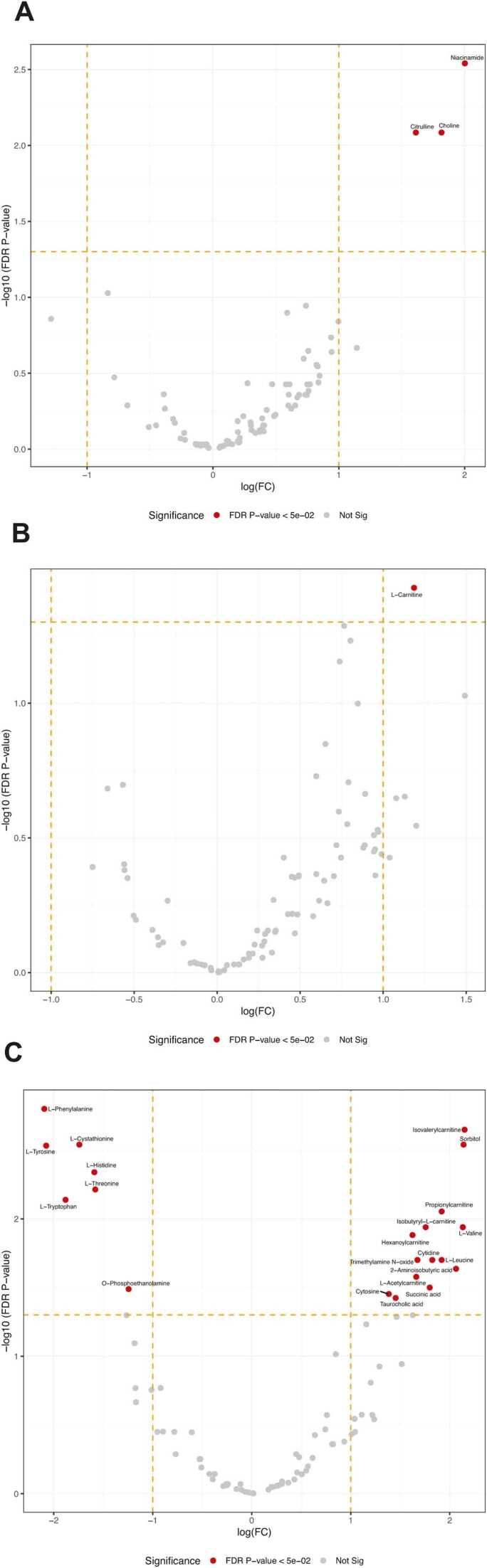
Comparison of single metabolite quantities in the CSF following (A) morphine, (B) oxaliplatin, and (C) streptozotocin treatment, compared with saline. Morphine (10 mg/kg) was administered twice daily for 14–16 days; the cumulative dose of oxaliplatin was 38 mg/kg during 4 weeks; the single dose of streptozotocin of 60 mg/kg was administered 4 weeks before sample extraction (n = 6–7 per group). Dotted line on y-axis delineates FDR = 0.05. Data-analysis with pairwise t-test.

**Fig. 10:**
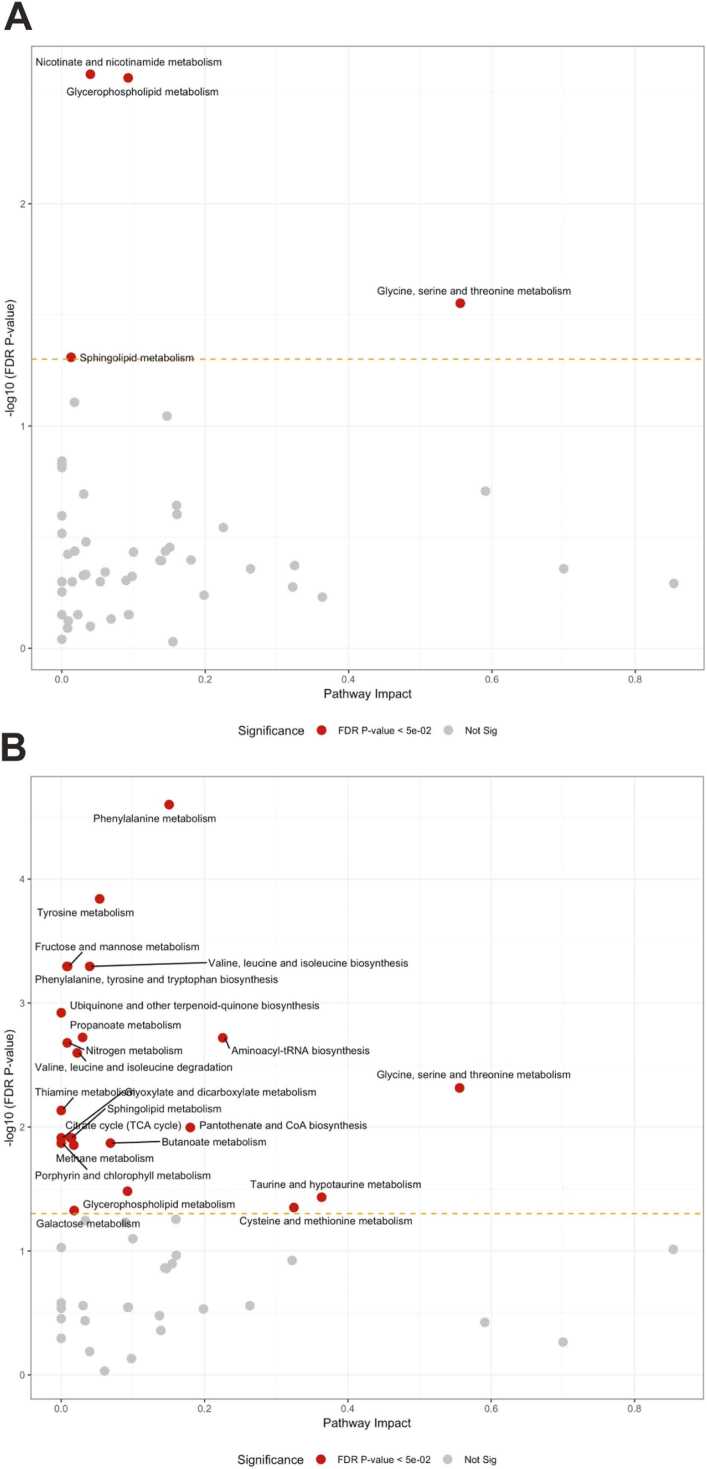
Pathway analyses of the metabolome in CSF following (A) morphine or (B) streptozotocin treatment, compared with saline. Morphine 10 mg/kg twice daily for 14 days; streptozotocin as a single dose of 60 mg/kg 4 weeks before sample extraction (n = 6–7 per group). Dotted line on y-axis delineates FDR = 0.05. Data-analysis by FIMM.

In the CSF samples, 102 metabolites were measured, of which 86 were detectable in at least 50% of the samples in each group. Three metabolites were altered in the morphine group: niacinamide, citrulline, and choline (Fig. 9A). In the oxaliplatin group, one metabolite, L-Carnitine (Fig. 9B), was altered while, in the STZ group, 21 were altered (Fig. 9C), of which the greatest changes were in L-Phenylalanine, L-Cystathionine, and L-Histidine. Additionally, pathway analyses of the data were carried out. In these, four were impacted by morphine, none by oxaliplatin, and 26 by streptozotocin (Fig. 10).

## 4. Discussion

In this study, we analysed the DRG transcriptome and CSF metabolome in rat models of opioid tolerance, oxaliplatin-induced and diabetic (streptozotocin-induced) polyneuropathies. Clear antinociceptive tolerance, signs of cold allodynia, hypoaesthesia and insulin-dependent diabetes developed in these groups, respectively. In the DRG, genes especially associated with nociception, inflammation, and glial cells were affected. Some genes, e.g. *Fkbp5 and Csf3r,* were similarly affected in all models, and some, e.g. *Alox15 and Slc12a5 and Gabrb1,* were differentially expressed in both diabetic and oxaliplatin rats, as discussed below. Several pain-related genes showed altered expression following the treatments. Below, we highlight some genes of special interest.

In the CSF metabolome, we demonstrate several changes in the diabetic rats, with the upregulation of nicotinamide in morphine-tolerant rats being especially interesting. To the best of our knowledge, the CSF metabolome has not previously been analysed in these conditions.

### 4.1. DRG transcriptome

Many genes in the DRG were differentially expressed in the oxaliplatin and diabetic groups, while only a few were differentially expressed in the morphine group. This is understandable as morphine may be used for extended periods, whereas oxaliplatin is a potent anticancer drug, with neuropathy being a common dose-limiting factor, further demonstrated by oxaliplatin’s causing abundant changes in pathways related to cell division (Cassidy and Misset, 2002). Similarly, untreated insulin-dependent diabetes is a lethal condition.

*Fkpb5*’s product, FK506 binding protein 5 (FKBP5), was upregulated in all groups. FKBP5 is upregulated by glucocorticoids but, in a negative feedback loop, it reduces the binding affinity of glucocorticoids to the receptor (Maiaru et al., 2016, Scharf et al., 2011). Interestingly, an FKBP5-antagonizing ligand has decreased hyperalgesia in several pain models (Maiaru et al., 2018). Additionally, silencing FKBP5 has been shown to alleviate NP at the spinal level (Yu et al., 2017). In the present study, we saw upregulation of FKBP5 in the DRG, suggesting that FKBP5 could also play a role in the development of NP at the DRG level. This supports the hypothesis that FKBP5 could be a target for treating NP.

*Csf3r* was downregulated in all groups: it codes for the receptor (G-CSFR) of granulocyte colony-stimulating factor (G-CSF), which is important in the production of granulocytes, such as neutrophiles (Mehta and Corey, 2021). G-CSF has been shown to alter gene expression in sensory nerves and to sensitize them (Bali et al., 2013, Stosser et al., 2011). However, it has also been shown to attenuate NP following peripheral nerve injury in rodents (Chao et al., 2012, Koda et al., 2014) and to have a nerve-preserving effect in diabetic neuropathy in rats (Lee et al., 2013). The different effects of G-CSF may reflect a dual role of inflammation in neuropathy development and repair.

The changes in *Fkpb5 and Csf3r* found in all three models may reflect common neuroinflammatory mechanisms in opioid tolerance and polyneuropathy, which could be of significance when treating NP with opioids.

These shared changes also raise the question of what effect chronic opioid administration might have on the transcriptome of dorsal root ganglia and other tissues in neuropathic animals, for example after oxaliplatin and streptozotocin treatment. This question would also be clinically relevant.

*Alox15*’s product, ALOX15 (arachidonate 15-lipoxygenase), upregulated in the oxaliplatin and diabetic groups, produces anti-inflammatory and antinociceptive resolvins and protectins (Lopez-Vicario et al., 2016, Romano et al., 2015; Russell and Schwarze, 2014), and, as such, is of interest. However, *Alox15* also plays a role in the regulation of adipose tissue and development of insulin resistance (Lieb et al., 2014), and a reduction in *Alox15* expression has been shown to improve neuropathy in mice with STZ-induced diabetes (Watcho et al., 2011). The role of the upregulation of ALOX15 in these models remains unclear.

The gene *Slc12a5* was strongly upregulated following oxaliplatin and STZ. It codes for the K-Cl co-transporter KCC2, which creates a chloride ion gradient needed for the inhibitory effects of GABAA receptors (Blaesse et al., 2009). KCC2 downregulation, in contrast to the upregulation we described in DRGs, is instrumental in the GABAergic disinhibition in NP in the spinal cord (Kitayama, 2018). *Gabrb1*, which was upregulated following oxaliplatin and STZ, codes for the beta-1 subunit of inhibitory GABAA receptors (Goetz et al., 2007). Therefore, upregulation of both *Gabrb1* and *Slc12a5* may enhance the inhibitory effects of GABA in neurons. However, KCC2 is not thought to be expressed in DRG neurons (Tan et al., 2020) and therefore the effect of KCC2 upregulation at the DRG level in NP is not clear. As such, it may reflect an unreported change in expression in glial cells of DRG, for example, and may be of significance in oxaliplatin-related neuropathy.

*Ptgds,* one of the few genes showing a large downregulation following morphine, codes for prostaglandin D2 synthase, an enzyme that converts prostaglandin H_2_ into prostaglandin D_2_ (Vane et al., 1998). Little is known about *Ptgds* or prostaglandin D2 in opioid tolerance or in the chronic use of opioids in general (Li et al., 2018). However, some studies suggest that NSAIDs, which inhibit the synthesis of prostaglandins, may increase opioid tolerance (Tsagareli et al., 2011). Therefore, this decrease in *Ptgds* may enhance opioid tolerance.

*Gal*, coding for the neuropeptide galanin (GAL), was downregulated in STZ-rats. GAL decreases neuronal hyperexcitability and is widely expressed in the nervous system (Mitsukawa et al., 2010). It has been described as having a neuroprotective role (Hobson et al., 2010, Liu and Hökfelt, 2002). In diabetic neuropathy, GAL has been shown to have antinociceptive effects through the GAL1 and GAL2 receptors (Yu et al., 2020). GAL also affects glucose metabolism, and its downregulation may also result from hyperglycaemia (Abot et al., 2018) and may be a specific contributor to diabetic neuropathy.

*Aqp4* codes for Aquaporin 4 (AQP4) and was upregulated in STZ-rats. AQP4 is a cell membrane water channel abundant in the astrocytes of the CNS, where it maintains cellular homeostasis and contributes to the glymphatic system (Halsey et al., 2018, Jung et al., 1994). Interestingly, changes in AQP4, have been proposed to have a role in nociception, especially in CNS astrocytes, perhaps by causing excess glutamate release and excitotoxicity (Nesic et al., 2005, Nesic et al., 2006, Verkman, 2005). Intriguingly, *Grin2a*, which was upregulated following STZ, codes for a subunit of NMDA receptors, which is important for NP (Deng et al., 2019, Zhou et al., 2011). The metabolic derangements in STZ-rats might cause excess release of glutamate and an increase in NMDA receptors, which might contribute to the neuropathy. NMDA antagonists have been studied as a treatment option for diabetic neuropathy, efficacy and safety still need to be demonstrated (Aiyer et al., 2018, Collins et al., 2010).

Additionally, *Gfap*, coding for glial fibrillary acid protein, a marker for satellite glial cells (SGCs) which play supporting roles in the DRG (Chen et al., 2022, Hanani, 2010), was upregulated following oxaliplatin, and downregulated after STZ. Increases in GFAP-staining have been observed in DRGs following oxaliplatin (Lee and Kim, 2020), in line with our findings. SGCs are activated in response to cytotoxic drugs and release several pro-inflammatory molecules: they have been shown to contribute to neuropathy and to NP (Brandolini et al., 2019, Villa et al., 2010, Warwick and Hanani, 2013). *Gfap* upregulation has been described in the DRG of rats two weeks after STZ administration (Hanani et al., 2014), while we demonstrate a downregulation after four weeks. Perhaps our finding reflects later reduced glial activity following a period of increased activity.

### 4.2. CSF metabolome

The most interesting finding in the metabolome of the CSF was that nicotinamide was upregulated four-fold after chronic morphine treatment. Nicotinate and nicotinamide metabolisms were also affected in pathway analyses after chronic morphine treatment. This pathway generates coenzymes such as NAD^+^ and NADP^+^ (nicotinamide adenine dinucleotide; NAD phosphate), crucial for redox reactions. Interestingly, coadministration of nicotinamide with morphine has been shown to attenuate morphine tolerance^8^. Furthermore, nicotinamide has been proposed to decrease opioid withdrawal symptoms through decreased nitric oxide synthase production (Pellat-Deceunynck et al., 1994; Toda et al., 2009). Increased NAD^+^ levels have been suggested to have theoretical benefits in addiction (Braidy et al., 2020). Nicotinamide has even been shown to exert an antinociceptive effect in rats in both inflammatory and NP models (Godin et al., 2011). As further support for the role of nicotinamide in opioid analgesia, tramadol has recently been shown to increase nicotinamide concentrations in the serum of mice (Jiang et al., 2021). Interestingly, a recent study reported that dezocine, a partial μ- and κ-opioid receptor agonist, inhibits the nicotinamide phosphoribosyltransferase (NAMPT) enzyme by directly binding to it, thus resulting in inhibition of conversion of nicotinamide into NAD^+^. Therefore, inhibition of NAMPT could increase nicotinamide but decrease NAD^+^ levels. If morphine had a similar effect, this could be a possible explanation for the increase of nicotinamide in this study (Xue et al., 2021). Although the mechanism of increase of nicotinamide by morphine is not known, the current finding may have clinical significance in opioid addiction and tolerance.

Levels of choline (a component of acetylcholine) were also increased after morphine treatment and it is noteworthy that nicotinamide has been shown to cause increases in choline levels in the CSF of rats (Vargas and Jenden, 1996); any connection between these findings remains to be studied.

In the oxaliplatin group, we demonstrated the levels of one metabolite, L-carnitine, to be increased. Carnitine is especially involved in regulating fatty acid beta-oxidation (Longo et al., 2016). Interestingly, acetyl-L-Carnitine has been shown to prevent some neurotoxicity caused by oxaliplatin (Ghirardi et al., 2005).

In the STZ group, we saw many changes in the CSF metabolome. In contrast to the other groups, we also saw several downregulated metabolites. One of particular interest was sorbitol, which showed increased concentrations. The polyol sorbitol is produced by the polyol pathway of carbohydrate metabolism (Garg and Gupta, 2022). In diabetes, sorbitol accumulation leads to osmotic stress and cell oedema, an important mechanism in chronic diabetic complications (Burg ans Kador, 1988). Increased CSF sorbitol levels (correlated with plasma glucose concentrations) have previously been described in diabetic neuropathy (Servo et al., 1977; Servo and Pitkänen, 1975).

## 4.3. Conclusions

In this study, we demonstrate abundant changes in the transcriptome of the DRGs in rats with diabetic and oxaliplatin-related neuropathies and fewer changes following chronic morphine treatment. Intriguingly, *Fkbp5 and Csf3r* changed in all models, which may reflect overlaps in their pathophysiologies. Several other changes related to nociception and inflammation are demonstrated, especially in the oxaliplatin and STZ groups. We also showed for the first time that the CSF metabolome is impacted in these models, and demonstrate changes in the CSF metabolome, particularly in the diabetic rats, and interestingly in the morphine group related to nicotinamide, known to be linked to opioid addiction and withdrawal. These changes are hypothesis-generating for future studies, drug targets, and perhaps better treatment of NP and opioid tolerance.

## Funding

Funding for this research was received from the 10.13039/501100000780European Union Seventh Framework Programme (FP7/2007–2013) (grant agreement no 602919) and 10.13039/100010135Finska Läkaresällskapet (Finnish Medical Society).

## CRediT authorship contribution statement

**Pekka V. Rauhala:** Conceptualization, Formal analysis, Funding acquisition, Investigation, Methodology, Project administration, Resources, Supervision, Writing – review & editing. **Kim J. Blomqvist:** Formal analysis, Investigation, Methodology, Writing – review & editing. **Tuomas O. Lilius:** Conceptualization, Investigation, Methodology, Writing – review & editing. **Eija A. Kalso:** Conceptualization, Formal analysis, Funding acquisition, Investigation, Methodology, Project administration, Resources, Supervision, Writing – review & editing. **Leena Karhinen:** Conceptualization, Writing – review & editing. **Vidya Velagapudi:** Data curation, Formal analysis, Investigation, Methodology, Writing – review & editing. **Fredrik Ahlström:** Conceptualization, Data curation, Formal analysis, Investigation, Methodology, Visualization, Writing – original draft, Writing – review & editing, Project administration. **Hanna Viisanen:** Formal analysis, Investigation, Methodology, Writing – review & editing.

## Declaration of competing interest

Eija Kalso has served on the advisory boards of Orion Pharma and Pfizer. The authors have no other conflicts of interest to disclose.

## Data Availability

Data will be made available upon request.
